# COVID-19 pandemic influence on self-reported health status and well-being in a society

**DOI:** 10.1038/s41598-022-12586-7

**Published:** 2022-05-24

**Authors:** Anna Moniuszko-Malinowska, Piotr Czupryna, Marlena Dubatówka, Magda Łapińska, Małgorzata Kazberuk, Aleksandra Szum-Jakubowska, Sebastian Sołomacha, Paweł Sowa, Łukasz Kiszkiel, Łukasz Szczerbiński, Anna Bukłaha, Piotr Paweł Laskowski, Karol Adam Kamiński

**Affiliations:** 1 Department of Infectious Diseases and Neuroinfections, Medical University of Bialystok, Zurawia 14, 15-540 Bialystok, Poland; 2 Department of Population Medicine and Lifestyle Diseases Prevention, Medical University of Bialystok, 15-269 Bialystok, Poland; 3 Medical University of Bialystok, Bialystok, Poland; 4 Society and Cognition Unit, University of Bialystok, 15-403 Bialystok, Poland; 5 Department of Endocrinology, Diabetology and Internal Medicine, Medical University of Bialystok, 15-276 Bialystok, Poland; 6 Clinical Research Centre, Medical University of Bialystok, Bialystok, Poland; 7 Department of Cardiology, University Hospital of Bialystok, 15-276 Bialystok, Podlaskie, Poland

**Keywords:** Environmental sciences, Medical research

## Abstract

To assess the frequency of persisting symptoms after SARS-CoV-2 infection and assessment of the effects of COVID-19 pandemic on selected parameters of self-reported health status and well-being half a year after the disease. The study population consisted of 3 groups: post-COVID-19 group I—172 patients; group II—172 patients with chronic disease, who have not suffered from COVID-19; group III—81 patients from a population study cohort—Bialystok PLUS. A standardized interview questionnaire was used to collect data in the three groups using the CATI (computer assisted telephone interviewing) technique. Interviews were conducted between October 2020 and January 2021, thus during the second wave of the pandemic in Poland. The subjective state of health in comparison with the state of health before the COVID-19 pandemic deteriorated in COVID-19 convalescents. Patients, who suffered from symptomatic COVID-19 were more prone to nervousness, anxiousness, tension than patients with oligosymptomatic course of the disease. Moreover, anxiety, fear and irritability were more frequent in Group I and II in comparison to Group III, whereas Group I and II did not differ significantly. The decrease in physical activity observed in COVID-19 patients mirrored the changes in general population. The most frequent persistent symptoms after COVID-19 are: general malaise, cough, smell and taste disorder, dyspnea. COVID-19 convalescents who experienced symptomatic disease are more prone to development of nervousness, anxiousness, tension and anxiety than patients with oligosymptomatic course of the disease. Females and younger patients who suffered from COVID-19 are more prone to development of mental distress than healthy population. No significant differences between COVID-19 convalescents and healthy population was observed as far as the attitude towards physical activity is concerned.

## Introduction

COVID-19 is a multisystem disease, which may lead to serious consequences, including death. There have been already 413 million cases registered worldwide, with 5.83 million of deaths^1^. Despite the fact that the disease has been known and diagnosed for only one year, loads of knowledge regarding the pathogenesis, clinical picture and diagnostics has been gained. Also more and more studies reveal the long-term sequelae of this disease, however the clinical trajectory and long-term outcomes for COVID-19 survivors are not fully known. Insight into potential infectious and postinfectious pathogenetic mechanisms linking SARSCoV2 to acute and long term neuropsychiatric complications is continuously updated. The direct and indirect psychological and social effects of the ongoing COVID19 pandemic, comprising the most prolonged global crisis since World War II is still unknown^2^.

Long term consequences of COVID-19 (known as ‘long COVID-19’) emerge as a chronic syndrome. It encompasses a plethora of debilitating symptoms (including shortness of breath, chest pain, palpitations and orthostatic hypotension) which can last for weeks or more following mild illness ^3^. A study conducted in Italy showed that, 53% of patients had fatigue, 43%—dyspnea and 22% were experiencing chest pain after 2 months^4^. Halpin et al. reported that after 4–8 weeks, persistent, chronic fatigue is present in more than two thirds of patients, followed by shortness of breath and symptoms of post-traumatic stress disorder^5^. In other single-center study of 143 patients recovering from COVID-19 in Italy, 44% of patients reported decreased quality of life and 87% of patients reported persistent symptoms including dyspnea, chest pain, cough, fatigue, and joint pain^6^.

Moreover, nowadays many patients suffer from psychological and psychiatric problems, similar to the ones already experienced during other pandemics such as Severe Acute Respiratory Syndrome (SARS) outbreak in 2003 and the earlier H1N1 outbreak in 1918. This concerns not only patients who recovered from the disease, but also those who were lock-downed for a long time. It is possible that COVID-19 pandemic may carry a second layer of psychological morbidity in the form of depression and mood disorder^7^. One should also take into consideration lifestyle changes due to isolation and lockdown that may affect the development of diseases in the future or deteriorate existing conditions. Therefore, the aim of our study was to assess the impact of pandemic on various aspects of local society lifestyle and mental health.

Aims: Assessment of the frequency of persisting symptoms after SARS-CoV-2 infection; assessment of the effects of COVID-19 pandemic on selected parameters of self-reported health status and well-being approximately half a year after the disease; comparison of the effects of COVID-19 pandemic on selected parameters of self-reported health status and well-being between COVID-19-convalescents, general population and people suffering from chronic diseases (patients with cardiovascular diseases) with no COVID-19 history.

## Methods

### Study population

The study population consisted of 3 groups:

Group I—Post-COVID-19 group—172 patients who had a positive SARS-CoV2 PCR test. This group was divided into Group Ia (symptomatic COVID-19—104 patients) and Group Ib (oligosymptomatic COVID-19—68 patients). These were patients hospitalized or treated in out-patients department because of COVID-19 and initially agreed to participate in the study. In this group, the response rate was 60%, where 172 interviews were successively completed out of 287 calls made.

Group II—172 patients with chronic disease represented by patients with coronary heart disease (a history of myocardial infarction or percutaneous coronary intervention (PCI)), who has not suffered from COVID-19 and initially agreed to participate in the study. The response rate here was 67%. Out of 257 records in the database, 172 questionnaire interviews were conducted successfully.

Group III—81 patients from a population study cohort—Bialystok PLUS. The Bialystok PLUS study describes the health of the local community by analysis of the examinations and questionnaires of a carefully selected cohort representative for the local population. These patients matched according to sex and age to COVID-19 group.

No exclusions criteria were used. Patients who agreed for participation were contacted.

The Bialystok PLUS study concerns the current health status of the population providing valuable information both about the development of the diseases but also about psychological and sociological backgrounds that may affect them^8^.

Symptomatic COVID-19 (Group Ia) was diagnosed if a patient experienced at least 3 symptoms out of 12 predefined (cough, dyspnea, fever, loss of smell and taste, weakness, etc.) during the acute phase of the disease.

Oligosymptomatic COVID-19 group (Group Ib) consisted of patients who experienced up to 2 symptoms during the acute phase of the disease.

A standardized interview questionnaire was used to collect data, which was carried out by trained staff conducting interviews on a daily basis as part of the Bialystok Plus cohort study and PhD students using the CATI (Computer Aided Telephone Interviewing) technique. In order to standardize and control the course of the telephone interview, LimeSurvey software was used, in which each respondent was given an individual token, which allowed for complete anonymity. An electronic script controlled the course of the survey, excluding the possibility of the interviewer making a mistake when moving between filtered questions. The entire survey consisted of 179 questions (including separately each statement from the battery, where respondents used, for example, the standardized Lickert scale for evaluation). However, the final number of questions was determined by the scenario, i.e. whether the respondent had the disease, was in quarantine, what and how many symptoms he/she had during and after the disease. The respondent had to answer a minimum of 49 questions and a maximum of 103 questions.

The survey was grouped into the following thematic blocks: I. Metrics, II. Health assessment, III. Symptoms of coronavirus in the household, IV. Testing for coronavirus, V. Mental well-being, VI. Feeling of loneliness, VII. Social distance and disinfection. VIII. Assessment of change in range of physical activity, VIII. Worries caused by the pandemic, IX. Alcohol and nicotine consumption, X. Lifestyle changes due to the pandemic. X. Symptoms during and after illness (among people who have undergone SARS-Cov2 virus infection).

Patients who answered that the symptoms were similar before and after pandemics were included to the “less often” category.

The study was conducted during the second wave of the pandemic—between October 2020 and January 2021.

### Statistical analysis

Descriptive statistics were presented as counts and frequencies for quantitative variable and as mean and standard deviation for continuous variable. Comparisons of variables between subgroups were conducted using the Chi^2 test for quantitative variable or Kruskall-Wallis test for continuous variable. Exact Fisher test was used when applicable. Z-test was used to determine whether predictor variables in logistic regression model have a significant effect. Statistical analysis was performed using Python Software Foundation (Version 3.9. Available at http://www.python.org) and STATA 16 (College Station, TX, USA). The criterion for statistical significance was set at p < 0.05.

### Ethics approval

The study was conducted according to the guidelines of the Declaration of Helsinki, and approved by the Bioethical Committee of Medical University of Bialystok, protocol code: APK.002.346.2020.

### Consent to participate

Informed consent was obtained from all individual participants included in the study.

### Consent to publish

The authors affirm that human research participants provided informed consent for publication.

## Results

### Selected clinical, sociological and psychological parameters in patients who have experienced COVID-19

The descriptive statistics with comparison of Groups are presented in Table 1a and 1b.

**Table 1:** Demographic data and descriptive statistics.

a							
Parameter		Group Ia (N = 104)	Group Ib (N = 68)	p-value			
Gender	Women	64 (61.5%)	34 (50.0%)	0.14			
	Men	40 (38.5%)	34 (50.0%)				
Age (mean ± SD)		48.3 ± 13.0	43.6 ± 12.3	0.03			
Residence	Village	25 (24.0%)	7 (10.3%)	0.02			
	City	79 (76.0%)	61 (89.7%)				

b							
Parameter		Group I	Group II	Group III	P I vs II	P I vs III	P II vs III
Gender	Women	98 (57.0%)	39 (22.7%)	42 (51.9%)	0.001	0.44	0.001
	Men	74 (43.0%)	133 (77.3%)	39 (48.1%)			
Age (mean ± SD)		46.4 ± 13.3	66.7 ± 8.0	47.8 ± 15.4	0.001	0.50	0.001

Thirty two out of 172 (18.6%) patients were hospitalized, while 140 did not require hospitalization.

The most frequent persisting symptoms after COVID-19 were: malaise – 58/172 (33.7%) – persisting for 30 days (min – 5 days, max—90 days), cough – 24/172 (13.9%) – persisting for 30 days (min – 7 days, max—90 days), dyspnea – 15/172 (8.7%) persisting for 25.5 days (min – 14 days, max – 60 days), smell and taste disorder – 31/172 (18%) – persisting for 21 days (min – 7 days, max -180 days), arrythmia 6/172 (3.4%) – persisting for 20 days (min – 10 days, max—30 days).

**Figure 1:**
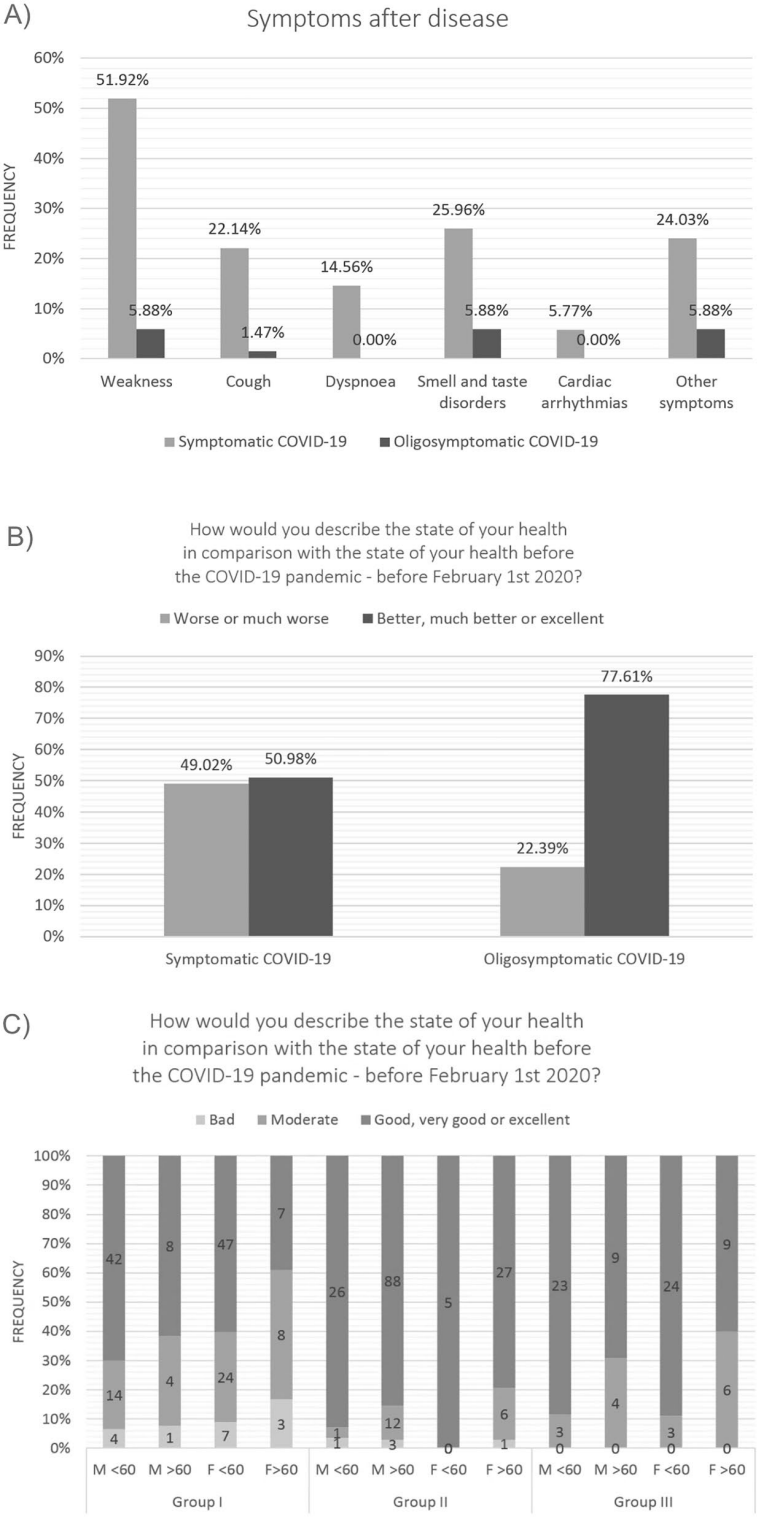
(**a**) Frequency of persisting symptoms after COVID-19 in the groups of symptomatic COVID-19 and oligosymptomatic COVID-19 patients. (**b**) Assessment of the subjective state of health in comparison with the state of health before the COVID-19 pandemic between Group Ia and Ib. (**c**) Assessment of the subjective state of health between post-COVID-19 patients with control group and group with chronic diseases in comparison with the state of health before the COVID-19 pandemic.

The comparison of Group Ia and Ib showed higher frequency of all reported psychological symptoms in Group Ia (Fig. 1). The subjective state of health in comparison with the state of health before the COVID-19 pandemic—before February 1st 2020 also differed between Group Ia and Ib (in Group Ia 50/102 (49%) and in Group Ib 15/67 (22.3%) reported deterioration of general well-being (p = 0.005)) (Fig. 1).

Psychological complications were also more frequent in the Group Ia (Table 2). 32% of patients from Group Ia and 13% of patients from Group Ib reported that after COVID-19 they were more prone to development of nervousness, anxiousness, tension and 18% of patients from Group Ia and 7% of patients from Group Ib were more prone to anxiety.

**Table 2:** Comparison of selected psychological parameters in patients who have experienced symptomatic COVID-19 (Group Ia) in comparison to patients who experienced oligosymptomatic COVID-19 (Group Ib).

Symptom		Group Ia (N = 104)	Group Ib (N = 68)	p-value
Feeling nervous, anxious or tense	More often	33 (32.7%)	9 (13.4%)	0.04
	Less often	68 (67.3%)	58 (86.6%)	
Feeling of fear as if something terrible might happen	More often	19 (18.8%)	5 (7.6%)	0.04
	Less often	82 (81.2%)	61 (92.4%)	
Rapid irritation or irritability	More often	13 (12.9%)	4 (6.1%)	0.15
	Less often	88 (87.1%)	62 (93.9%)	
In comparison to the time before the coronavirus pandemic, you drink alcohol	More often	2 (2.6%)	3 (5.7%)	0.42
	Less often	74 (97.4%)	50 (94.3%)	
In comparison to the time before the coronavirus pandemic, you smoke	More often	1 (11.1%)	1 (10%)	0.94
	Less often	8 (88.9%)	9 (90%)	

The analysis revealed that age and gender had no impact frequency of psychological complications, while comparison of symptoms based on place of inhabitance showed that city inhabitants are more prone to anxiety development that country residents (p < 0.05) (Table 3).

**Table 3:** Comparison of selected psychological parameters in patients who have experienced symptomatic COVID-19 (Group I) when considering the sex, age and place of living.

Symptom		Women (N = 98)	Men (N = 74)	p-value	Age < 60 years (N = 141)	Age ≥ 60 years (N = 31)	p-value	City (N = 140)	Village (N = 32)	p-value
Feeling nervous, anxious or tense	More often	29 (30.2%)	13 (18.1%)	0.07	36 (26.3%)	6 (19.4%)	0.42	33 (24.1%)	9 (29.0%)	0.57
	Less often	67 (69.8%)	59 (81.9%)		101 (73.7%)	25 (80.6%)		104 (75.9%)	22 (71.0%)	
Feeling of fear as if something terrible might happen	More often	11 (11.6%)	6 (8.3%)	0.49	13 (9.6%)	4 (12.9%)	0.49	17 (12.5%)	0 (0%)	0.04
	Less often	84 (88.4%)	66 (91.7%)		123 (90.4%)	27 (87.1%)		119 (87.5%)	31 (100%)	
Rapid irritation or irritability	More often	16 (16.8%)	8 (11.1%)	0.3	21 (15.4%)	3 (9.7%)	0.41	21 (15.4%)	3 (9.7%)	0.41
	Less often	79 (83.2%)	64 (88.9%)		115 (84.6%)	28 (90.3%)		115 (84.6%)	28 (90.3%)	
In comparison to the time before the coronavirus pandemic, you drink alcohol	More often	3 (4.5%)	2 (3.2%)	0.69	4 (3.7%)	1 (4.8%)	0.82	4 (3.8%)	1 (4.3%)	0.9
	Less often	63 (95.5%)	61 (96.8%)		104 (96.3%)	20 (95.2%)		102 (96.2%)	22 (95.7%)	
In comparison to the time before the coronavirus pandemic, you smoke	More often	2 (25%)	0 (0%)	0.08	2 (12.5%)	0 (0%)	0.52	2 (13.3%)	0 (0%)	0.44
	Less often	6 (75%)	11 (100%)		14 (87.5%)	3 (100%)		13 (86.7%)	4 (100%)	

### Selected clinical, sociological and psychological parameters in patients who have experienced COVID-19 in comparison to with coronary heart disease patients and Bialystok Plus Group

#### Comparison of the whole study group

Comparison of post-COVID-19 patients with control group and group with chronic diseases showed significant differences in the subjective state of health in comparison with the state of health before the COVID-19 pandemic—before February 1st 2020 also differed between Group I, II and III (Fig. 1).

Anxiety, fear and irritability were more frequent in Group I and II in comparison to Group III, whereas Group I and II did not differ significantly (Table 4).

**Table 4:** Comparison of selected psychological parameters in patients who have experienced COVID-19 (Group I) in comparison to with coronary heart disease patients (Group II) and Bialystok Plus Group (Group III).

Symptom		Group I	Group II	Group III	P I vs II	P I vs III	P II vs III
Feeling nervous, anxious or tense	More often	42 (25%)	37 (21.6%)	8 (9.9%)	0.46	0.01	0.02
	Less often	126 (75%)	134 (78.4%)	73 (90.1%)			
Feeling of fear as if something terrible might happen	More often	24 (14.4%)	26 (15.2%)	2 (2.5%)	0.83	0.01	0.01
	Less often	143 (85.6%)	145 (84.8%)	79 (97.5%)			
Rapid irritation or irritability	More often	17 (10.2%)	25 (15.5%)	1 (1.3%)	0.22	0.01	0.01
	Less often	150 (89.8%)	146 (84.5%)	78 (98.7%)			
In comparison to the time before the coronavirus pandemic, you drink alcohol	More often	2 (10.5%)	2 (7.4%)	2 (14.3%)	0.71	0.74	0.48
	Less often	17 (89.5%)	25 (92.6%)	12 (85.7%)			
In comparison to the time before the coronavirus pandemic, you smoke	More often	5 (3.9%)	12 (9.4%)	4 (5.6%)	0.08	0.57	0.35
	Less often	124 (96.1%)	116 (90.6%)	67 (94.4%)			

Physical activity of patients from 3 analyzed groups differed. Various kind of activities, such as physical activity at work, domestic activities (e.g. washing, cleaning, cooking), sports activities (e.g. jogging, sports cycling, strength training), recreational activities (e.g. walking, gardening) were analyzed (Table 5).

**Table 5:** Comparison of selected physical activity parameters in patients who have experienced COVID-19 (Group I) in comparison to with coronary heart disease patients (Group II) and Bialystok Plus Group (Group III).

Symptom		Group I	Group II	Group III	P I vs II	P I vs III	P II vs III
Physical activity at work (also at home office) decrease	More often	29 (18.3%)	11 (8%)	14 (22.2%)	0.01	0.51	0.04
	Less often	129 (81.7%)	126 (92%)	49 (77.8%)			
Domestic activities (e.g. washing, cleaning, cooking) decrease	More often	24 (14.2%)	9 (5.2%)	8 (9.9%)	0.005	0.34	0.12
	Less often	145 (85.8%)	162 (94.8%)	73 (90.1%)			
Sports activities (e.g. jogging, sports cycling, strength training) decrease	More often	75 (44.9%)	21 (12.4%)	36 (48%)	0.00001	0.59	0.000001
	Less often	92 (55.1%)	148 (87.6%)	38 (52%)			
Recreational activities (e.g. walking, gardening decrease	More often	68 (40.2%)	31 (18.1%)	34 (42%)	0.00001	0.79	0.0001
	Less often	101 (59.8%)	140 (81.9%)	47 (58%)			
Mobility activities (e.g. walking, cycling on the way to work / shopping) decrease	More often	65 (62.5%)	43 (25.4%)	25 (31.6%)	0.01	0.30	0.31
	Less often	104 (37.5%)	126 (74.6%)	54 (68.4%)			

Although there was a decrease in physical activity observed in COVID-19 patients it mirrored the changes in general population (no significant differences between Group I and III were observed).

Patients from Group II presented the most active lifestyle and pandemic did not seem to influence it.

#### Comparison between the groups I, II and III when considering the age and sex

The division of patients based on the age showed that younger patients (< 60 years old) with COVID-19 were more prone to fear and anxiety than patients from Group III, while in older patients there were no significant differences observed (Table 6).

**Table 6:** Comparison between the groups when considering the age and sex.

Symptom		Group I	Group II	Group III	P I vs II	P I vs III	P II vs III
**Male N = 74 + 133 + 39 = 246**							
Feeling nervous, anxious or tense	More often	13 (18.1%)	27 (20.5%)	4 (10.3%)	0.68	0.28	0.15
	Less often	59 (81.9%)	105 (79.5%)	35 (89.7%)			
Feeling of fear as if something terrible might happen	More often	6 (8.3%)	17 (12.9%)	0 (0%)	0.33	0.06	0.02
	Less often	66 (91.7%)	115 (87.1%)	39 (100%)			
Rapid irritation or irritability	More often	8 (11.1%)	21 (15.9%)	2 (5.1%)	0.35	0.29	0.01
	Less often	64 (88.9%)	111 (84.1%)	37 (94.9%)			
In comparison to the time before the coronavirus pandemic, you smoke	More often	0 (0%)	10 (9.3%)	0 (0%)	0.29	–	0.4
	Less often	11 (100%)	98 (90.7%)	7 (100%)			
In comparison to the time before the coronavirus pandemic, you drink alcohol	More often	2 (3.2%)	2 (9.1%)	2 (5.9%)	0.26	0.52	0.65
	Less often	61 (96.8%)	20 (90.9%)	32 (94.1%)			
**Female N = 98 + 39 + 42 = 179**							
Feeling nervous, anxious or tense	More often	29 (30.2%)	10 (25.6%)	4 (9.5%)	0.6	0.01	0.06
	Less often	67 (69.8%)	29 (74.4%)	38 (90.5%)			
Feeling of fear as if something terrible might happen	More often	11 (11.6%)	8 (20.5%)	1 (2.5%)	0.18	0.09	0.01
	Less often	84 (88.4%)	31 (79.5%)	39 (97.5%)			
Rapid irritation or irritability	More often	16 (16.8%)	5 (12.8%)	0 (0%)	0.56	0.01	0.02
	Less often	79 (83.2%)	34 (87.2%)	42 (100%)			
In comparison to the time before the coronavirus pandemic, you smoke	More often	2 (25%)	0 (0%)	2 (28.6%)	0.22	0.88	0.19
	Less often	6 (75%)	5 (100%)	5 (71.4%)			
In comparison to the time before the coronavirus pandemic, you drink alcohol	More often	3 (4.5%)	2 (10%)	2 (5.4%)	0.36	0.85	0.52
	Less often	63 (95.5%)	18 (90%)	35 (94.6%)			
**Age < 60 years N = 141 + 33 + 53 = 227**							
Feeling nervous, anxious or tense	More often	36 (26.3%)	6 (18.2%)	4 (7.5%)	0.33	0.04	0.13
	Less often	101 (73.7%)	27 (81.8%)	49 (92.5%)			
Feeling of fear as if something terrible might happen	More often	13 (9.6%)	4 (12.1%)	1 (1.9%)	0.66	0.07	0.04
	Less often	123 (90.4%)	29 (87.9%)	52 (98.1%)			
Rapid irritation or irritability	More often	21 (15.4%)	4 (12.1%)	1 (1.9%)	0.63	0.01	0.04
	Less often	115 (84.6%)	29 (87.9%)	52 (98.1%)			
In comparison to the time before the coronavirus pandemic, you smoke	More often	2 (12.5%)	1 (11.1%)	2 (15.4%)	0.92	0.82	0.77
	Less often	14 (87.5%)	8 (88.9%)	11 (84.6%)			
In comparison to the time before the coronavirus pandemic, you drink alcohol	More often	4 (3.7%)	4 (14.3%)	2 (4.4%)	0.03	0.83	0.14
	Less often	104 (96.3%)	24 (85.7%)	43 (95.4%)			
**Age ≥ 60 years N = 31 + 139 + 28 = 198**							
Feeling nervous, anxious or tense	More often	6 (19.4%)	31(22.5%)	4 (14.3%)	0.71	0.6	0.33
	Less often	25 (80.6%)	107 (77.5%)	24 (85.7%)			
Feeling of fear as if something terrible might happen	More often	4 (12.9%)	21 (15.2%)	0 (0%)	0.74	0.06	0.03
	Less often	27 (87.1%)	117 (84.8%)	26 (100%)			
Rapid irritation or irritability	More often	3 (9.7%)	22 (15.9%)	1 (3.6%)	0.37	0.35	0.08
	Less often	28 (90.3%)	116 (84.1%)	27 (96.4%)			
In comparison to the time before the coronavirus pandemic, you smoke	More often	0 (0%)	1 (5.6%)	0 (0%)	0.68	–	0.81
	Less often	3 (100%)	17 (94.4%)	1 (100%)			
In comparison to the time before the coronavirus pandemic, you drink alcohol	More often	1 (4.8%)	8 (8%)	2 (7.7%)	0.61	0.68	0.96
	Less often	20 (95.2%)	92 (92%)	24 (92.3%)			

Also anxiety and fear were significantly more frequent in Group I than in Group III in females than males (Table 6).

The tendency to smoke and drink alcohol differed only between the group I and II when considering drinking (Table 6). Many patients declined to answer the questions concerning their habits, so results must be interpreted with care.

#### Multivariate logistic regression analysis

Multivariate logistic regression with sex and age adjustment showed, that in symptomatic patients, that risk of feeling nervous, anxious or tense and rapid irritation or irritability was 3.2 and 3.26 time higher, respectively (Table 7, 8).

**Table 7:** Univariate analysis variables in Group Ia and Ib.

	Feeling nervous, anxious or tense			Rapid irritation or irritability			Feeling of fear		
	OR	p	95% CI	OR	p	95% CI	OR	p	95% CI
Group	3.13	0.006	1.38–7.07	2.83	0.05	1.00–7.99	2.29	0.164	0.71–7.35

**Table 8:** Multivariate analysis of analyzed variables in Group Ia and Ib.

	Feeling nervous, anxious or tense			Rapid irritation or irritability			Feeling of fear		
	OR	p	95% CI	OR	p	95% CI	OR	p	95% CI
Group	3.20	0.007	1.37–7.44	3.26	0.030	1.12–9.47	2.15	0.205	0.66–7.05
Gender	1.81	0.129	0.84–3.87	1.49	0.406	0.58–3.78	1.31	0.622	0.45–3.77
Age*	0.53	0.221	0.19–1.46	0.44	0.218	0.12–1.63	1.24	0.730	0.37–4.17
Place of residence	1.03	0.956	0.41–2.56	2.32	0.209	0.62–8.60	–	–	–

* As binary value ≤ 60 years old and > 60 years old.

Comparison of patients from Group II and III with Group I also showed no significant influence of age and sex of the results while the OR of nervousness, irritability and fear were lower in Group III than in Group I (0.33; 0.15 and 0.11, respectively).

Patients from Group I were more eager to perform physical activity at home, recreational activities (e.g. walking, gardening decrease, sports activities (e.g. jogging, sports cycling, strength training), mobility activities (e.g. walking, cycling on the way to work / shopping) than patients from Group II.

The willingness of activity at home and mobility was lower in patients over 60 years (OR—0.27 and 0.47 respectively) (Tables 9, 10).

**Table 9:** Univariate analysis of variables (Group I as comparison group).

	Feeling nervous, anxious or tense			Rapid irritation or irritability			Feeling of fear			Alcohol			Smoking			Physical activity at work			Domestic activities			Recreational activities			Sports activities			Mobility activities		
	OR	p	95% CI	OR	p	95% CI	OR	p	95% CI	OR	p	95% CI	OR	p	95% CI	OR	p	95% CI	OR	p	95% CI	OR	p	95% CI	OR	p	95% CI	OR	p	95% CI
Group II	1.21	0.464	0.73–2.00	0.94	0.923	0.51–1.71	0.66	0.218	0.34–1.28	0.39	0.085	0.13–1.14	1.47	0.713	0.19–11.48	0.39	0.012	0.39–0.81	0.34	0.007	0.15–0.75	0.33	0.000	0.20–0.54	0.17	0.10–0.30	3.32–9.95	0.55	0.011	0.34–0.87
Group III	0.33	0.007	0.15–0.47	0.15	0.012	0.03–0.66	0.11	0.036	0.01- 0.87	1.48	0.568	0.38–5.70	1.42	0.744	0.17- 11.51	0.80	0.548	0.39- 1.64	1.51	0.341	0.65–3.53	0.93	0.794	0.54–1.59	0.86	0.591	0.90–1.49	1.35	0.299	0.77–2.38

**Table 10:** Multivariate analysis of variables (Group I as comparison group).

	Feeling nervous, anxious or tense			Rapid irritation or irritability			Feeling of fear			Alcohol			Smoking			Physical activity at work			Domestic activities			Recreational activities			Sports activities			Mobility activities		
	OR	p	95% CI	OR	p	95% CI	OR	p	95% CI	OR	p	95% CI	OR	p	95% CI	OR	p	95% CI	OR	p	95% CI	OR	p	95% CI	OR	p	95% CI	OR	p	95% CI
Group II	1.06	0.860	0.55–2.05	0.91	0.818	0.41–2.02	0.63	0.296	0.26–1.51	0.33	0.097	0.09–1.22	0.27	0.391	0.01–5.30	0.45	0.085	0.18–1.11	0.15	0.001	0.06–0.40	0.28	0.001	0.15–0.51	0.16	0.000	0.08–0.32	0.37	0.001	0.20–0.68
Group III	0.33	0.008	0.15–0.75	0.15	0.012	0.03–0.66	0.11	0.036	0.01–0.87	1.53	0.538	0.39–5.99	1.24	0.847	0.14–11.33	0.79	0.528	0.38–1.63	1.99	0.130	0.82–4.85	0.98	0.947	0.57–1.69	0.87	0.624	0.50–1.52	1.52	0.161	0.85–2.73
Gender	1.54	0.096	0.93–2.58	1.04	0.899	0.47- 2.06	1.67	0.149	0.83–3.33	1.14	0.805	0.41–3.17	7.91	0.084	0.76–82.25	1.27	0.450	0.68–2.36	0.89	0.747	0.45–1.78	0.80	0.311	0.51–1.24	1.03	0.901	0.65–1.64	0.78	0.253	0.50–1.20
Age*	1.04	0.906	0.57–1.88	0.98	0.956	0.47- 2.06	1.22	0.632	0.54–2.78	0.83	0.727	0.29–2.39	0.14	0.199	0.00–2.84	1.47	0.348	0.66–3.27	0.27	0.001	0.12–0.57	0.67	0.140	0.40–1.14	0.93	0.802	0.54–1.61	0.47	0.006	0.28–0.80

*As binary value ≤ 60 years old and > 60 years old.

^#^ Statistically significant values (*p* < 0.05).

## Discussion

Our study uses data gathered in BIALYSTOK Plus study, which is a large database concerning population health of Podlaskie Region inhabitants. More and more reports underline the role of long-COVID-19, what is especially important from the population medicine point of view. The multisystem nature of Long-COVID compared to previously studied post-acute sequelae of human coronaviruses has raised questions about how to most effectively identify indicators of Long-COVID. An analysis of 32 symptoms in patients with and without SARS-CoV-2 infection identified several symptoms that were more frequent in patients with COVID-19 in comparison to other illnesses of comparable severity. In systematic review that included at least 100 patients; based on the 15 studies that met the inclusion criteria, the authors identified 55 symptoms of Long-COVID. They reported that the five most common symptoms evaluated in the literature were fatigue, headache, attention disorder, hair loss, and dyspnea^9–11^. It is known, that high rates of post-traumatic stress disorder (PTSD) are evident among patients that have been hospitalised because of COVID-19.^12^ In our study the most frequent symptoms reported by COVID-19 convalescents were: general malaise, cough, smell and taste disorder, dyspnea. The spectrum of post-COVID-19 symptoms may differ depending the characteristic of patients included in the study, as ours covered mostly non-hospitalised COVID-19 patients. Worth emphasizing in our study is observation regarding the differences of psychological consequences between patients with oligosymptomatic versus symptomatic disease. COVID-19 convalescents who experienced symptomatic disease are more prone to development of nervousness, anxiousness, tension and anxiety than patients with oligosymptomatic course of the disease.

In our study, we examined not only symptoms of long-COVID-19, but also the impact of the COVID-19 pandemic on the mental health of various groups of patients with different background: who suffered from COVID-19, patients with cardiovascular diseases and healthy population exposed to risk of the infection with SARS-CoV-2. Although adolescents and young people have lower risk of severe COVID-19 development, hospitalisation and sequelae due to the disease, the pandemic is having important effects on the mental health of young population^13^. Our study showed, that females and younger convalescents are more prone to development of mental distress after COVID-19. This is in line with results of other studies, as it was observed that in developed countries younger age and female sex associated with greater levels of anxiety, stress and depression^14–17^.

The vulnerability of younger people to these conditions may be partially explained by the fact that older adults may have more experience in coping with stress. It was also previously reported, that physical isolation from family members or loved ones during quarantine or hospital stay can produce psychological instability among people with COVID-19^18^. Other potential pathomechanism of mental disorders development might be caused by the coronavirus affecting the brain directly or indirectly by inducing a massive cytokine response harming the brain^19^. On the other hand, it is well known that when negative mood state persists over time it can result in the dysregulation of physiological response involved in the regulation of the immune system^20^. Thus, a significant potential exists for the psychological harm inflicted by the pandemic to translate into physical harm. This could include an increased susceptibility to the virus, worse outcomes if infected, worse responses to vaccinations.

Our findings add new information to data from literature and they suggest that there is considerable potential to develop interventions to mitigate the mental health effects of the pandemic and a role for public health sector, which could simultaneously reduce the risk of COVID-19 infection as well as help to manage some of the concomitant psychological distress. Moreover, measuring awareness and anxiety of the patients are essential for prevention strategy planning, and identifying risk groups. This knowledge may be useful for physicians, who can better understand if the patients have a rise in various symptoms caused by COVID-19. The psychosomatic effects of the anxiety caused by the pandemic should be kept in mind by clinicians as patients might require a psychiatric consultation during the treatment^21^. This applies not only to patients who suffered from COVID-19, but also other members of society, as pandemic has impact on everybody.

Physical activity is an important part of well-being, so we also assessed the influence of pandemic on this issue. Healthy behaviours such as exercise have been found to be protective factors for poorer mental health during the first 10 weeks of the pandemic. The benefits of exercise are well documented in the literature on mental health. Exercise is known to improve cerebral blood flow, sleep, mental alertness, self-esteem and energy, and prevent social withdrawal^22,23^. It may also provide a distraction from daily challenges.

Worthy of discussion are our observations regarding patients with cardiovascular diseases, who presented with the least changes in physical activity and that pandemic has affected it in a relatively lower degree when comparing to other groups. Probably these patients are more disciplined and accustomed to physical exercises. They are more educated of the role of exercise in their diseases and they focus on how to get it done, whereas the general population avoids contact and exercise, and they are probably busy with work.

No significant differences between post-COVID-19 patients and healthy population was observed as far as the physical activity is concerned.

It was already stated, by Jia et al. that smoking and alcohol consumption were also associated with greater anxiety during the COVID-19 pandemic^14^. It has been widely documented that people with poor mental health are more likely to engage in unhealthy behaviors such as smoking and drinking alcohol^24^. In our cohort, no differences were observed regarding neither in alcohol nor in smoking frequency increase, however data may be biased, as many patients have not responded to questions regarding addictions.

Our findings highlight a number of issues worthy of discussion, but we also need to acknowledge several limitations, such as possible under-reporting due to stigma associated with mental health and unhealthy habits, despite piloting and validation, as well as possible bias in self-reported experiences of pandemic-related stressors due to feelings of nervousness, anxiety or irritability. Additional limitations caused by the use of a retrospective question, in our article about subjective health change, are well described by Hipp et al., where the authors note that respondents' ratings of their pre-pandemic conditions were positively correlated with changes in their current conditions, the difference in change between past and present is likely to be underestimated, and aggregate estimates may still be biased if changing social norms bring about collective changes in answering behaviours (e.g., washing hands etc.)^25^. As a result of the analysis, respondents were more likely to report more negative pre-pandemic conditions (T0) in a later interview (T2) if they experienced worsening during an ongoing pandemic.

The survey was conducted in Białystok, a city of 300 000 inhabitants in eastern Poland, in Podlaskie Region, which at the time of the survey, during the second wave of the pandemic, had significantly higher incidence rates than other large cities in Poland. Moreover, the timing of the study coincided with a period of reintroduced restrictions and lockdown after the summer respite following the first wave of the pandemic. Based on research conducted by the Centre for Public Opinion Research on a representative sample of Poles, we can conclude that in the same period, epidemic restrictions were mostly observed, i.e. social distance (79%), wearing masks (78%) and washing hands more often (94%)^26^. Moreover, this was a period when there was still no prospect of a vaccine being developed, the third wave of the pandemic was forecast for spring 2021, and access to health care was severely limited. Outpatient clinics operated mostly by tele-consultation, and treatments at specialists were postponed.

Plotting the socio-cultural background in the period when the surveys were conducted, based on representative research conducted by the authors of the article at the beginning of the second wave of the pandemic in Poland (March 2021—a year after the announcement of the pandemic in Poland), where retrospective questions were also used, it is worth noting the perceived social impact of the pandemic, which may have indirectly affected psycho-social indicators^27^. The national survey asked about the limitations of activities previously performed regularly and to what extent the decrease in their frequency bothered respondents. It turns out that after the first year of the pandemic, many aspects of social life ceased (e.g., concert attendance, cultural life—cinemas, museums, theaters, restaurants, pubs, domestic and international tourism, meetings with friends) and, more importantly, it bothered respondents a lot.

Summing up, our study delivers important information regarding influence of pandemic on general well-being, but is based on relatively small sample size, so further studies are needed.

## Conclusions

1. The most frequent persistent symptoms after COVID 19 are: general malaise, cough, smell and taste disorder, dyspnea.
2. COVID-19 convalescents who experienced symptomatic disease are more prone to development of nervousness, anxiousness, tension and anxiety than patients with oligosymptomatic course of the disease.
3. In COVID-19 convalescents there are no differences between age and sex groups regarding psychological disorders frequency.
4. Females and younger patients who suffered from COVID-19 are more prone to development of mental distress after the disease than healthy population.
5. No significant differences between COVID-19 convalescents and healthy population was observed as far as the attitude towards physical activity is concerned.
