# Author Correction: COVID-19 pandemic influence on self-reported health status and well-being in a society

**DOI:** 10.1038/s41598-023-42895-4

**Published:** 2023-09-19

**Authors:** Anna Moniuszko-Malinowska, Piotr Czupryna, Marlena Dubatówka, Magda Łapińska, Małgorzata Kazberuk, Aleksandra Szum-Jakubowska, Sebastian Sołomacha, Paweł Sowa, Łukasz Kiszkiel, Łukasz Szczerbiński, Anna Bukłaha, Piotr Paweł Laskowski, Karol Adam Kamiński

**Affiliations:** 1 Department of Infectious Diseases and Neuroinfections, Medical University of Bialystok, Zurawia 14, 15-540 Bialystok, Poland; 2 Department of Population Medicine and Lifestyle Diseases Prevention, Medical University of Bialystok, 15-269 Bialystok, Poland; 3 Medical University of Bialystok, Bialystok, Poland; 4 Society and Cognition Unit, University of Bialystok, 15-403 Bialystok, Poland; 5 Department of Endocrinology, Diabetology and Internal Medicine, Medical University of Bialystok, 15-276 Bialystok, Poland; 6 Clinical Research Centre, Medical University of Bialystok, Bialystok, Poland; 7 Department of Cardiology, University Hospital of Bialystok, 15-276 Bialystok, Podlaskie, Poland

Correction to: *Scientific Reports* 10.1038/s41598-022-12586-7, published online 24 May 2022

The original version of this Article contained an error in the Funding section.

“This research was funded by National Centre of Science grant UMO-2020/37/B/NZ7/03380 within call OPUS-19.”

now reads:

"This research was funded by the National Science Centre, Poland grant within the program OPUS-19 (grant number 2020/37/B/NZ7/03380)."

The original Article has been corrected.

